# Preoperative Management of Perihilar Cholangiocarcinoma

**DOI:** 10.3390/cancers14092119

**Published:** 2022-04-24

**Authors:** Ryan J. Ellis, Kevin C. Soares, William R. Jarnagin

**Affiliations:** 1 Hepatopancreatobiliary Service, Department of Surgery, Memorial Sloan Kettering Cancer Center, New York, NY 10065, USA; ellisr1@mskcc.org (R.J.E.); soaresk@mskcc.org (K.C.S.); 2 Department of Surgery, Weill Cornell Medical College, New York, NY 10021, USA

**Keywords:** cholangiocarcinoma, hilar cholangiocarcinoma, hepatectomy, preoperative management, biliary drainage

## Abstract

**Simple Summary:**

In patients diagnosed with potentially resectable perihilar cholangiocarcinoma, deliberate and coordinated preoperative workup and optimization of the patient and future liver remnant are crucial. Strategic optimization and multidisciplinary evaluation may reduce the rate of unnecessary procedures, perioperative complications, and non-therapeutic laparotomy for unresectable disease. In this review, we describe preoperative assessment and optimization of patients with perihilar cholangiocarcinoma, including imaging workup, evaluation of resectability, preoperative drainage and hypertrophy-inducing procedures, and the role of diagnostic laparoscopy.

**Abstract:**

Perihilar cholangiocarcinoma is a rare hepatobiliary malignancy that requires thoughtful, multidisciplinary evaluation in the preoperative setting to ensure optimal patient outcomes. Comprehensive preoperative imaging, including multiphase CT angiography and some form of cholangiographic assessment, is key to assessing resectability. While many staging systems exist, the Blumgart staging system provides the most useful combination of resectability assessment and prognostic information for use in the preoperative setting. Once resectability is confirmed, volumetric analysis should be performed. Upfront resection without biliary drainage or portal venous embolization may be considered in patients without cholangitis and an estimated functional liver remnant (FLR) > 40%. In patients with FLR < 40%, judicious use of biliary drainage is advised, with the goal of selective biliary drainage of the functional liver remnant. Percutaneous biliary drainage may avoid inadvertent contamination of the contralateral biliary tree and associated infectious complications, though the relative effectiveness of percutaneous and endoscopic techniques is an ongoing area of study and debate. Patients with low FLR also require intervention to induce hypertrophy, most commonly portal venous embolization, in an effort to reduce the rate of postoperative liver failure. Even with extensive preoperative workup, many patients will be found to have metastatic disease at exploration and diagnostic laparoscopy may reduce the rate of non-therapeutic laparotomy. Management of perihilar cholangiocarcinoma continues to evolve, with ongoing efforts to improve preoperative liver hypertrophy and to further define the role of transplantation in disease management.

## 1. Introduction

Cholangiocarcinoma is a rare gastrointestinal malignancy, occurring at a rate of three cases per 100,000 people [1]. Approximately 40% of cholangiocarcinomas are intrahepatic, with the remaining 60% arising from the extrahepatic biliary tree. The incidence of cholangiocarcinoma is increasing over time, with a measured increase of 1.8% per year for extrahepatic cholangiocarcinoma [2].

Unfortunately, patients with cholangiocarcinoma often present with locally advanced or metastatic disease, and only 44% have localized disease at presentation [3]. Among those with localized disease, even fewer have tumors that are anatomically resectable owing to a high rate of local invasion and proximity to the major hepatic vasculature. For the minority of patients that present with resectable local disease, resection and transplantation offer the only opportunity for cure. Resection remains the primary surgical therapy due to both organ scarcity and unclear survival benefit of transplant compared to conventional resection. Resection is potentially curative and requires aggressive surgical strategies in experienced hepatopancreatobiliary (HPB) centers to assure negative margins in well selected candidates. Recurrence rates, however, remain high [4].

Initial reports of short-term operative outcomes following resection of perihilar cholangiocarcinoma were very poor. In the original series of perihilar resections reported by Klatskin, 12 of 13 patients suffered perioperative mortality [5]. Outcomes improved significantly through the 1990s, with perioperative mortality rates dropping to less than 5% for hepatectomy overall and 10% for perihilar resections [6,7]. Further improvement has been demonstrated in contemporary series, owing to improved patient selection and perioperative management techniques [8,9].

The complication profile of hepatectomy for hilar cholangiocarcinoma is driven by post-hepatectomy liver failure [10], which is primarily due to infection of the biliary tract and inadequate liver volume [11]. As such, the key to improved perioperative outcomes is preoperative optimization of the functional liver remnant to avoid postoperative liver insufficiency and failure [12]. The major principles to be outlined in this review are preprocedural imaging analysis for assessment of resectability, targeted biliary drainage based on the proposed resection, and volumetric optimization.

## 2. Preoperative Imaging

Abdominal ultrasound is often the first imaging modality performed given its low cost and accessibility. Findings are non-specific and consist of biliary dilatation with low sensitivity in predicting the level of biliary obstruction [13]. The initial staging workup includes both abdominal and chest imaging and is focused on identifying patients with disseminated disease, including intrahepatic metastatic disease. Although conventional computed tomography (CT) can provide valuable information about staging and regional lymphadenopathy, it is suboptimal in assessing vascular involvement and tumor resectability [14].

Imaging assessment to determine resectability requires an intimate understanding of the tumor location in relation to the portal structures. Resectability is determined by assessing the following criteria: extent of biliary involvement, vascular involvement, lobar atrophy, and evidence of metastatic disease. This is best accomplished with high-resolution CT angiography of the liver, including the chest and pelvis and/or magnetic resonance imaging/magnetic resonance cholangiopancreatography (MRI/MRCP). Both studies are usually performed since they provide complimentary information. Biliary stents may substantially reduce the diagnostic and staging accuracy of even the highest-quality scans due to biliary decompression and imaging artifact. Perhaps more importantly, biliary decompression performed before the disease extent is fully defined often results in malpositioned stents that may complicate management or even make surgical intervention impossible. As such, every effort should be made to obtain high-quality imaging prior to biliary intervention.

CT imaging should be performed with thin cuts with arterial and portal venous phases. Our preferred protocol is a multiphase CT angiogram with thin (1 mm) cuts including early arterial, late arterial, portal venous, and delayed phases. A recent meta-analysis demonstrated a pooled sensitivity of 89% and specificity of 92% for portal vein involvement, as well as 84% sensitivity and 95% specificity for hepatic artery involvement for similar CT protocols [15]. CT imaging provides less precise estimation of the longitudinal extent of the tumor within the biliary tract [16]. This is the particular strength of contrast-enhanced MRI/MRCP, which provides more accurate characterization of the biliary tree [17]. In experienced hands, duplex US can also be a useful diagnostic modality for assessing vascular involvement. In a single-center series of 63 patients, duplex US predicted portal vein involvement in 93% of cases, with a specificity of 99% and a positive predictive value of 97% [18]. With adequate cross-sectional evaluation and expert assessment of resectability, invasive cholangiography (e.g., endoscopic or percutaneous cholangiography) is rarely indicated for diagnostic purposes.

The role of positron-emission tomography (PET) CT in hilar cholangiocarcinoma is not as firmly established. A recent systematic review and meta-analysis assessing the diagnostic accuracy of PET CT demonstrated a sensitivity and specificity for diagnosis of the primary tumor of 91.7% and 51.3%, respectively. PET CT also performed with high sensitivity and moderate specificity for lymph node involvement (88.4% and 69.1%, respectively), with high sensitivity and improved specificity in identification of distant metastases (85.4% and 89.7%, respectively) [19]. Routine use of PET CT is yet to be investigated in prospective trials, and at this time is best used to further evaluate equivocal findings of other cross-sectional modalities.

**Figure 1:**
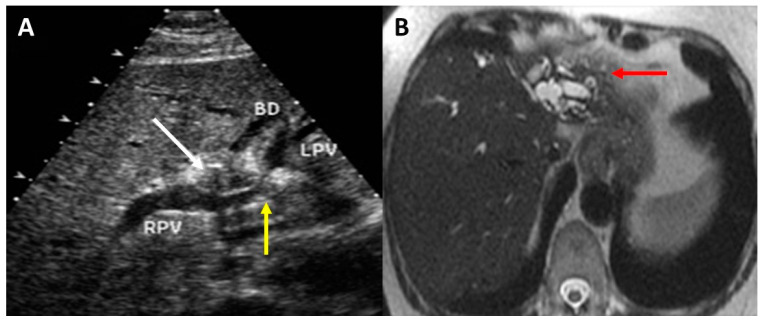
Lobar atrophy associated with perihilar cholangiocarcinoma. Preoperative ultrasonography (**A**) demonstrating perihilar biliary mass (**white arrow**) with resultant narrowing of the left portal vein (**yellow arrow**). MRI (**B**) demonstrated substantial left lobar atrophy (**red arrow**).

There are important caveats in the accuracy and interpretation of preoperative imaging. Even the highest-quality imaging often fails to identify an obvious mass. Instead, the pattern of biliary ductal dilation, hepatic lobar atrophy, and hypertrophy often localize the tumor. Obstructed bile ducts are dilated up to the level of the tumor. Longstanding biliary obstruction can lead to lobar atrophy and indicate extent of tumor involvement. Lobar atrophy is pronounced when both ipsilateral portal vein and biliary obstruction occur. Cross-sectional imaging shows an atrophic lobe of the liver with dilated and crowded bile ducts and hypertrophy of the contralateral lobe (Figure 1). The right hepatic artery typically courses posterior to the common hepatic duct therefore involvement of the right hepatic artery in a left-sided biliary tumor requiring left liver resection precludes resection or requires hepatic artery reconstruction in select cases. Lymph node assessment on cross-sectional imaging lacks sensitivity and specificity but can be assessed with esophagogastroduodenoscopy/endoscopic ultrasonographic (EGD/EUS) biopsy in cases with high index of suspicion. Finally, a dilated gallbladder is generally inconsistent with a diagnosis of hilar cholangiocarcinoma and should raise the possibility of an alternative diagnosis, such as gallbladder/cystic duct cancer or a tumor involving the more distal bile duct.

Imaging alone is insufficient in preoperative assessment of the patient. Comprehensive preoperative assessment is required, and information such as tumor markers, inflammatory markers (e.g., IgG4), liver function tests and performance status may aid in identifying patients at high risk of occult advanced or metastatic disease or excessive morbidity [20]. This includes ruling out a benign etiology for a perihilar mass or stricture, such as those observed in IgG4-related sclerosing cholangitis [21]. Retrospective series have noted that between 3% and 15% of resected hilar strictures are benign on final pathology [21,22]. This has led to increased interest in more aggressive tissue sampling, such as via SpyGlass endoscopic evaluation of the biliary tree [23]. Despite advances in preoperative diagnosis, it remains difficult to differentiate between benign and malignant lesions without resection. Moreover, repeated attempts at biopsy can increase complication rates and delay resection. In cases with high index of suspicion for autoimmune cholangiopathy, serum IgG4 levels can be helpful and a trial of steroids is reasonable. When a malignant diagnosis is confirmed preoperatively, upfront resection is preferred in resectable disease without nodal involvement [24]. Neoadjuvant chemotherapy and/or chemoradiotherapy in locally advanced cases may downstage selected patients rendering them appropriate for surgical resection [25].

## 3. Assessment of Resectability and Staging

After imaging evaluation, assessment of resectability and accurate preoperative staging are critical for both surgical planning and treatment sequencing. There are multiple staging systems with varying degrees of detail in anatomic assessment of the primary tumor. The modified Bismuth–Corlette system focuses solely on the level of biliary involvement and classifies patients based solely on this variable [26]. Type I Bismuth–Corlette tumors are distal to the biliary confluence and involve the common hepatic duct; Type II tumors involve the confluence with minimal extension into either the right or left hepatic duct; Type III tumors extend past the biliary confluence into either the right (IIIA) or left (IIIB) hepatic duct to the level of the secondary biliary radicals; Type IV tumors involve the confluence and bilateral hepatic ducts to the level of the secondary biliary radicles. While the Bismuth–Corlette system provides valuable information in planning biliary reconstruction, it is limited as a true staging system by its lack of assessment of the portal vasculature and unclear relationship with overall prognosis. Similarly, the 8th edition of the American Joint Commission for Cancer (AJCC) tumor-node-metastasis staging system provides stages for perihilar cholangiocarcinoma after resection based on pathology but does not evaluate the anatomic configuration of the tumor and therefore has no utility in preoperative management.

Given these limitations, a staging system developed at Memorial Sloan Kettering Cancer Center (MSKCC) was designed to address resectability and prognosis [27,28]. This classification scheme, along with subsequent revisions [7], provides a comprehensive assessment of perihilar tumors (Table 1). Using this system, resectability was well stratified based on T stage (59% of T1, 31% of T2, and 0% of T3) [7]. These findings were subsequently validated externally by Matsuo and colleagues, who demonstrated 64% resectability in T1 tumors, 41% in T2, and 1.3% in T3 [29]. However, it should be noted that approximately 20% of patients are found to be unresectable at the time of exploration even with modern preoperative imaging [7].

Resectability and extent of hepatectomy are determined by assessing four criteria: (1) extent of biliary tree involvement, (2) lobar atrophy, (3) vascular involvement and (4) the presence of metastatic disease Adequate assessment of resectability is critical not only to ensure oncologic success, but also to technically plan the hepatectomy. The relationship of the biliary mass to the hepatic inflow at the hilum often dictates the extent of hepatectomy and is best assessed by high-quality cross-sectional imaging performed prior to biliary decompression as detailed above. The right hepatic artery typically courses posterior to the common bile duct; therefore, involvement of the right hepatic artery in a left-sided tumor precludes resection or requires right hepatic artery reconstruction. Assessment of liver atrophy indicates extent of biliary and vascular involvement. Long-standing biliary obstruction causes moderate atrophy, whereas ipsilateral biliary and vascular involvement leads to significant atrophy characterized by crowded biliary ducts in a hypoperfused, shrunken liver and contralateral liver hypertrophy.

## 4. Principles of Biliary Drainage

Biliary obstruction is a hallmark of perihilar cholangiocarcinoma, and identification of the level of biliary obstruction, combined with appropriately planned biliary drainage, is a critical component of the initial work up. It should be emphasized that ill-advised biliary stenting, based on incomplete information, can have far-reaching consequences that can impact on subsequent therapy. While patients presenting with cholangitis clearly require expeditious drainage, there continues to be significant debate regarding additional indications for biliary drainage and the optimal method of drainage once pursued. Routine biliary drainage has been advocated by many authors with a goal of reaching a bilirubin below 3 mg/dL, driven by the concept that robust drainage improves liver regeneration and improve postoperative outcomes [30]. This viewpoint is not universally supported, and multiple subsequent studies have demonstrated either no benefit and perhaps worse outcomes when patients with predicted future liver remnant of at least 30 to 50% are subjected to preoperative drainage [12,31,32,33]. An analysis of sixty patients after resection of perihilar cholangiocarcinoma by Kennedy et al. demonstrated equivalent postoperative outcomes in patients with FLR ≥ 30% with or without preoperative biliary drainage. In contrast, patients with FLR < 30% that did not undergo preoperative biliary drainage had a significantly higher rate of hepatic insufficiency (33%) compared to those with preoperative drainage (0%) [31]. These findings were reinforced in subsequent series, including work by Wiggers et al. demonstrating increased mortality after preoperative drainage in patients with FLR > 50% (12% vs. 0%) [12]. Based on these two studies, patients with functional liver remnant > 40% without cholangitis may forego routine drainage [34].

The rationale for selective biliary drainage is supported by the concept that biliary instrumentation and misplaced biliary drains can lead to significant infectious complications and could preclude resection [35]. The mechanism underlying this association is complex and likely multifactorial, but is driven by seeding of the biliary tract leading to perioperative cholangitis [36]. In a series of 133 patients published by Ribero et al., the rate of cholangitis was 41% in patients undergoing preoperative biliary drainage compared to 6% in those without drainage, and cholangitis was associated with both post-hepatectomy liver failure and perioperative mortality [11]. These risks of biliary drainage are exacerbated by failed attempts requiring multiple interventions, which is more likely when the initial attempt at decompression is performed at inexperienced centers or endoscopically [37,38]. Moreover, biliary contamination has been associated with the development of resistant organisms that may worsen postoperative infectious complications [39]. Some authors have advocated utilization of empiric broad-spectrum antibiotics in patients with a history of biliary instrumentation for hilar obstruction [40].

There are three primary methods for obtaining biliary drainage: percutaneous transhepatic biliary drainage (PTBD), endoscopic biliary drainage (EBD), and endoscopic nasobiliary drainage (ENBD). PTBD was the primary option historically prior to the rise of EBD, which has since supplanted PTBD as the most commonly performed biliary decompression procedure in the United States [41]. This has been driven primarily by increased comfort and experience with EBD in both benign and malignant settings, as well as concerns about the nuisance PTBD may be to patients [42]. The short-term morbidity and mortality of the two procedures are the same at high-volume institutions, but outcomes of both may be dependent on facility-level procedural volumes and local expertise [41].

**Figure 2:**
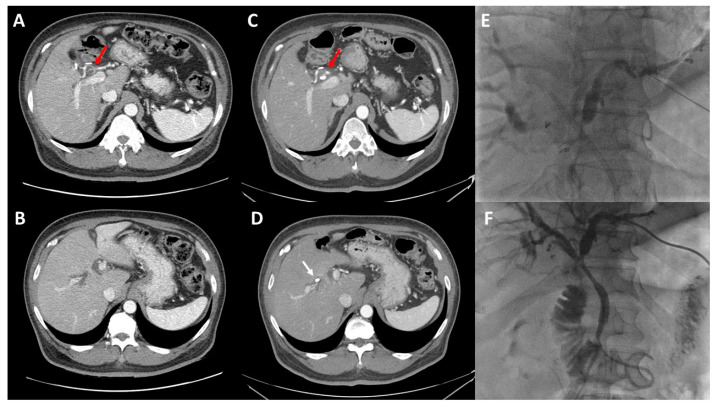
Repeat drainage following misplaced endoscopic biliary drain. Computed tomography showing perihilar tumor with involvement of right hepatic artery ((**A**), **red arrow**) with dilation of both left and right intrahepatic biliary trees (**B**). Repeat scan after placement of endoscopic biliary stent shows persistent arterial involvement ((**C**), **red arrow**) with inappropriate placement of the endoscopic stent into the right liver ((**D**), **white arrow**). The left liver (future liver remnant) remains undrained. Patient subsequently underwent selective percutaneous access of the left biliary tree (**E**) with placement of an internal-external biliary drain (**F**).

While EBD is the most common biliary decompression technique overall, it has significant shortcomings in the management of perihilar cholangiocarcinoma. Endoscopic drainage requires a sphincterotomy and traversing the contaminated intestinal tract during stent placement, leading to bacterial seeding of the biliary tree. Indeed, EBD has been demonstrated in multiple series and meta-analyses to be associated with higher rates of perioperative cholangitis [43]. EBD also has a higher failure rate due to the blind nature of stent placement and often requires additional PTBD [38]. In a series of 288 patients undergoing EBD for perihilar cholangiocarcinoma, 38% required subsequent PTBD, most often those with marked hyperbilirubinemia and left-sided obstructions [44]. The combination of potentially worsened biliary seeding and higher rates of repeat interventions likely underlies the observed higher rates of cholangitis following EBD. In contrast, PTBD has lower rates of infectious complications and allows for highly selective placement into sectional or segmental ducts for targeted drainage (Figure 2) [43]. Outcomes of PTBD can be further optimized using techniques that leave the drainage catheter past the level of biliary obstruction but above the Sphincter of Oddi, which has been associated with improved 90-day stent patency and reduced infectious stent complications when compared with trans-sphincteric percutaneous drainage [45].

There have been concerns raised about the oncologic sequelae of PTBD, specifically with regard to seeding of drainage tracks. Komaya et al. reported a series of 320 patients with significantly higher rates of seeding metastases at one year following PTBD (19.7%) compared to EBD (3.5%) [46]. However, it should be noted that this series defined seeding metastases not only as catheter tract disease but also as isolated right-sided peritoneal or pleural disease. Subsequent studies have not demonstrated higher rates of seeding metastases following PTBD, and early locoregional recurrences are likely markers of metastatic disease rather than drainage catheter related oncologic complications [47,48].

Due to the ongoing controversy regarding the best approach to biliary drainage, Coelen and colleagues performed a randomized controlled trial in the Netherlands comparing PTBD with EBD [38]. The primary endpoint of the trial was significant preoperative complications, and the trial enrolled a total of 54 patients from 2013 to 2016 of a preplanned 106 patients. Unfortunately, the study was stopped early due to higher observed postoperative mortality in the PTBD group (41%) compared to the EBD group (11%). However, these results must be interpreted with significant caution. First, the difference in overall mortality was driven by factors likely unrelated to drainage in the PTBD group, including cardiac events, progression of disease, and surgical mortality. The preoperative complication rates between the PTBD and EBD groups were no different (67% vs. 63%). Moreover, consistent with previous observational data, the conversion rate from EBD to PTBD (56%) was significantly higher than conversion from PTBD to EBD (4%). It is difficult to ascribe the differential mortality observed to the method of biliary drainage, and the results may be due to Type I error given the low accrual of the study prior to termination. Additional efforts to provide prospective data to guide biliary drainage practice have been difficult, including early termination of the INTERCPT trial due to poor accrual [49].

While PTBD and EBD are the most common modalities of biliary drainage in the West, endonasal biliary drainage (ENBD) is used commonly in the East, with proponents citing more durable drainage and fewer complications than percutaneous approaches. In one study by Kawakubo and colleagues, ENBD was associated with lower risk of biliary reintervention [50]. A larger, multicenter retrospective review of 374 patients with malignant perihilar biliary obstruction showed no differences between EBD and ENBD as the initial drainage technique in patients with perihilar cholangiocarcinoma [51].

**Figure 3:**
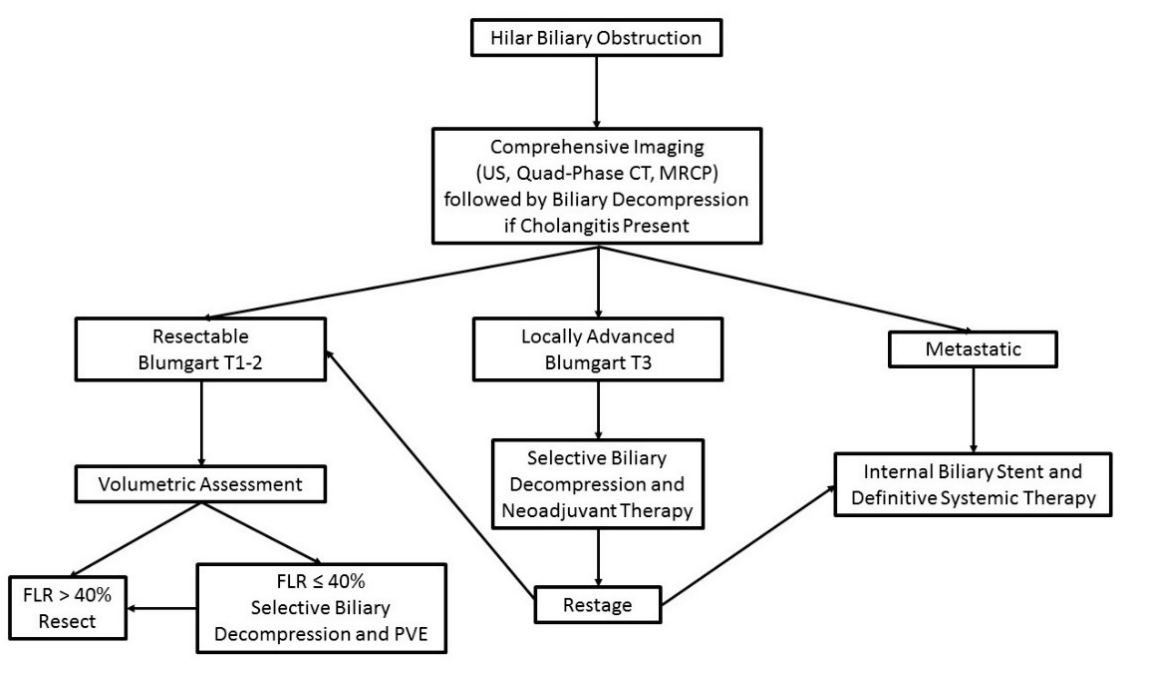
Algorithm for preoperative assessment and management of patients with perihilar cholangiocarcinoma.

Taken together, these data help to define an effective strategy for preoperative biliary drainage in perihilar cholangiocarcinoma that is preferred by the authors (Figure 3). First, early input from an experienced HPB surgeon is critical to optimize drainage and avoid unnecessary procedures. In the absence of cholangitis and in patients with adequate liver volumes, resection can be attempted without drainage. Second, when drainage is indicated, it should be performed in a targeted fashion, with the goal to minimize contamination of the biliary tree and repeat procedures. Instrumentation of obstructed biliary ducts without subsequent drainage is associated with a high incidence of cholangitis [52]. Thus, the authors prefer selective PTBD into the future liver remnant. This strategy allows for decompression and metabolic normalization of the FLR without contaminating the obstructed, contralateral liver.

## 5. Volumetric Analysis and Optimization

Post-hepatectomy liver failure is the most feared and devastating complication of major hepatectomy for any indication and is closely related to the volume and quality of the functional liver remaining after resection [11,12]. Along with planning preoperative biliary drainage, calculation and optimization of the functional liver remnant (FLR) are the second major considerations in perioperative optimization of patients undergoing resection for perihilar cholangiocarcinoma.

**Figure 4:**
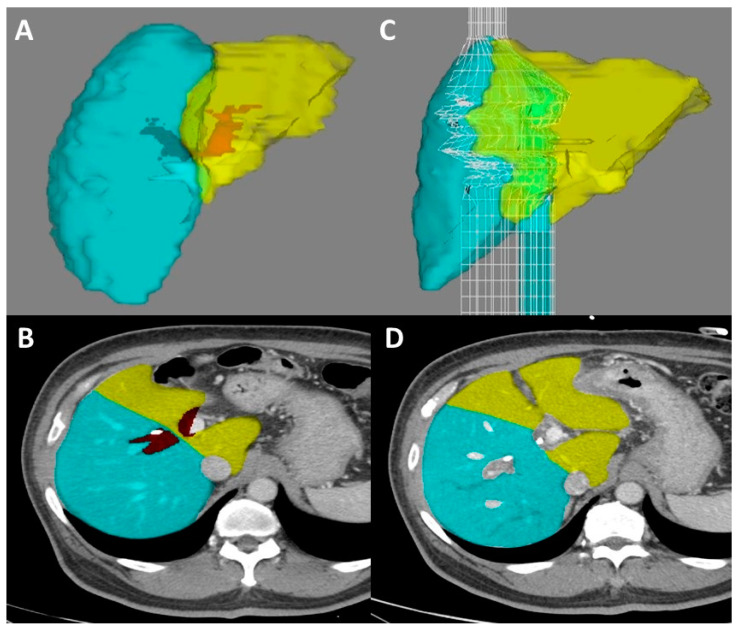
Preoperative and postoperative volumetric analysis with interval portal vein embolization. Computed tomography-based volumetric analysis (TeraRecon Inc., Durham, NC, USA) with perihilar cholangiocarcinoma. Prior to portal vein embolization (panels (**A**,**B**)), functional liver remnant after planned right hepatectomy was calculated to be 31.9% (yellow). Patient underwent right portal vein embolization without embolization of Segment IV. After three weeks, repeat volumetry was performed (panels (**C**,**D**)). The newly calculated FLR (yellow) was 45.9%, yielding a KGR of 4.7%.

Assessment of liver volume and calculation of FLR are well-established practices that should be considered routine for any major hepatectomy, especially in patients with underlying liver disease or previous hepatotoxic chemotherapy [53,54,55]. Briefly, multiple software applications are available that allow the surgeon to define what sections of the liver will be included in the specimen and which will remain. This allows for calculation of the FLR, reported as a percentage of the preoperative liver volume in the patient (Figure 4). The safe cutoff for resection varies based on the study and health of the underlying liver, but is often considered 25% for a healthy liver, 30% in those previously treated with hepatotoxic chemotherapy, and 40% in patients with substantial cholestasis or cirrhosis [1,56,57]. Due to the high rates of biliary stasis in perihilar cholangiocarcinoma, we suggest a conservative percentage cutoff of 40% prior to consideration of portal venous embolization to improve the FLR.

Portal vein embolization (PVE), first described by Makuuchi in 1990, has become a mainstay of therapy and critical to preoperative optimization in liver surgery [58]. The complication profile of the procedure is favorable, most commonly including low-grade fever or gastrointestinal distress and occurring in less than 20% of patients [59]. Major bleeding or thrombotic complications are rare. PVE leads to contralateral lobar hypertrophy over the course of 3 weeks and has been associated with decreased liver failure and mortality in perihilar cholangiocarcinoma resections [9,60]. Along with the absolute volume of the FLR, kinetic growth rate (KGR) has emerged as a key predictor of perioperative morbidity and mortality allowing for a dynamic assessment of liver hypertrophy [61]. KGR is defined as the growth in the FLR divided by the weeks since PVE, and is often presented as %/week. Following PVE, a KGR of 2.66%/week has been associated with 0% rates of postoperative liver failure and mortality [62]. In contrast to the FLR volume alone, which does not provide information on the function of the underlying liver, a KGR of at least 2.66%/week is a good indicator of liver health and regenerative capacity and thus is an integral component of preoperative assessment. Higher postoperative morbidity should be anticipated in patients undergoing hepatectomy with a KGR below this threshold.

More aggressive approaches to induce liver hypertrophy, including association of liver partition and portal vein ligation for staged hepatectomy (ALPPS) [63] and liver venous deprivation (LVD), have been explored [64]. ALPPS has been shown to yield fast liver hypertrophy and allows for definitive resection in as little as 7–10 days, but requires a second operation and has been associated with prohibitively high 90-day operative mortality in perihilar cholangiocarcinoma (20–48%) [65,66]. Olthof and colleagues compared outcomes of 29 ALPPS patients from the international ALPPS registry with 29 standard resection patients based on similar future liver remnant volume [66]. The mortality rate in the ALPPS group was 48% compared to 24% in patients without ALPPS. The median survival was 6 months in the ALPPS group versus 29 months in the matched control group. Given these results, ALPPS currently has no role in management of perihilar cholangiocarcinoma [67]. LVD, which adds hepatic venous embolization to traditional PVE, has also shown promise in promoting more robust liver hypertrophy with a more acceptable complication profile. The earliest LVD series were conducted using staggered portal venous and hepatic venous embolization and did not provide a significant temporal benefit over PVE alone [64]. More contemporary attempts at LVD include simultaneous embolization of the portal and hepatic venous systems and are associated with more robust hypertrophy, but require additional study prior to being incorporated into the treatment algorithm for perihilar cholangiocarcinoma [68,69].

## 6. Diagnostic Laparoscopy

Once a patient has undergone complete workup and preoperative liver optimization, consideration should be given to diagnostic laparoscopy. As previously discussed, even with modern imaging techniques the rate of unresectable disease on exploration is as high as 20–50% [7,70]. In series from the UK [71] and MSKCC [72], patients with radiographically resectable perihilar cholangiocarcinoma were found to have metastatic disease in 27% and 25% of cases, respectively. Yield is even higher for patients with T2/T3 tumors, where it may be as high as 36%. A recent meta-analysis implied that this yield may be decreasing with time, possibly due to ongoing improvements in cross-sectional imaging [73]. Overall, it is the authors opinion that the yield and opportunity to avoid a non-therapeutic laparotomy make routine use of staging laparoscopy a reasonable strategy, especially in patients with more locally advanced disease.

## 7. Consideration of Liver Transplant

Preoperative evaluation of patients with perihilar cholangiocarcinoma should include an understanding of the evolving role of liver transplantation in disease management. Initial results of orthotopic liver transplantation in unresectable perihilar cholangiocarcinoma were very poor, with high recurrence rates and two-year survival below 50% [74]. There has recently been renewed interest in transplantation for highly selected patients, particularly those with underlying primary sclerosing cholangitis (PSC).

The Mayo Clinic has developed a rigorous protocol for patients being considered for transplantation [75]. Eligible patients must have radiographic evidence of a mass lesion <3 cm at the biliary stricture, with either endoluminal tissue diagnosis or CA 19–9 > 100 U/mL. Patients are excluded from consideration if they have nodal or metastatic disease, have previously undergone attempted resection or transperitoneal biopsy, have had previous malignancy, or if they are unable to complete neoadjuvant therapy [76]. Patients meeting these criteria then undergo neoadjuvant chemotherapy and radiation followed by operative assessment of abdominal disease burden. In a report of 90 patients that underwent transplant after this protocol, the 5-year survival was 75% [77]. Subsequent analysis from the Mayo clinic comparing patients undergoing transplant (*n* = 90) and standard resection (*n* = 124) demonstrated equivalent survival between the groups after adjusting for age, lymph node status, and tumor size [78]. Another retrospective review by the US Extrahepatic Biliary Malignancy Consortium actually demonstrated improved 5-year survival in patients undergoing transplant compared to resection (64% vs. 18%, *p* < 0.001) [79].

Despite this encouraging improvement in outcomes, transplant is only appropriate for a small subset of patients with perihilar cholangiocarcinoma, primarily those with underlying liver disease due to PSC or with localized tumors with extensive biliary and/or vascular involvement. The ongoing TRANSPHIL prospective, randomized trial (NCT02232932) will help to further establish the role of transplantation. At this time, patients with resectable disease continue to be treated primarily with standard resection while consideration of transplantation is reserved for patients with locally advanced disease or disease in a background of primary sclerosing cholangitis [1].

## 8. Conclusions

Preoperative evaluation of patients with perihilar cholangiocarcinoma begins with thorough imaging assessment of the biliary anatomy, including the relationship between the tumor and major hepatic vasculature. In patients with resectable disease without cholangitis, drainage should be deferred until thorough surgical planning has occurred in order to maximize the quality of imaging and allow for thoughtful drainage focusing on the FLR. Percutaneous drainage has the advantage of being more targeted to segments that will be in the FLR, minimizing instrumentation and risks of contamination. Avoiding drainage of the diseased segment of liver further reduces unnecessary contamination. In resectable patients with an insufficient FLR, PVE can provide hypertrophy necessary for resection and is the preferred volumetric optimization procedure. Even after extensive workup, rates of unresectability are high and diagnostic laparoscopy can be considered to avoid some non-therapeutic laparotomies. Patients with locally unresectable tumors, with no evidence of metastatic disease, and particularly those with underlying PSC or other liver parenchymal disease, require prompt referral to transplants centers for complete evaluation. Appropriate use of adjunctive preoperative interventions at experienced centers with multidisciplinary involvement may improve outcomes.

## Figures and Tables

**Table 1:** Perihilar Cholangiocarcinoma Blumgart T Stage Criteria.

Stage	Definition
T1	Tumor involving confluence with or without unilateral extension to second-order biliary radicles AND No evidence of vascular involvement or hepatic atrophy
T2	Tumor involving confluence with or without unilateral extension to second-order biliary radicles AND Ipsilateral portal vein involvement and/or ipsilateral hepatic lobar atrophy
T3	Tumor involving confluence with bilateral extension to second-order biliary radicles OR Unilateral extension into second-order biliary radicles with contralateral portal vein involvement and/or hepatic lobar atrophy OR Main/bilateral portal vein involvement
